# Larvicidal activity of *Acacia nilotica* extracts against *Culex pipiens* and their suggested mode of action by molecular simulation docking

**DOI:** 10.1038/s41598-024-56690-2

**Published:** 2024-03-15

**Authors:** Mohamed M. Baz, Nancy M. El-Shourbagy, Abeer Mousa Alkhaibari, Hattan S. Gattan, Mohammed H. Alruhaili, Abdelfattah Selim, Ibrahim Taha Radwan

**Affiliations:** 1 Entomology Department, Faculty of Science, Benha University, Benha, 13518 Egypt; 2 Department of Animal Medicine (Infectious Diseases), Faculty of Veterinary Medicine, Benha University, Toukh, 13736 Egypt; 3 Department of Biology, Faculty of Science, University of Tabuk, 71491 Tabuk, Saudi Arabia; 4 Department of Medical Laboratory Sciences, Faculty of Applied Medical Sciences, King Abdulaziz University, Jeddah, Saudi Arabia; 5 Special Infectious Agents Unit, King Fahad Medical Research Center, King AbdulAziz University, Jeddah, Saudi Arabia; 6 Department of Clinical Microbiology and Immunology, Faculty of Medicine, King AbdulAziz University, Jeddah, Saudi Arabia; 7 Supplementary General Sciences Department, Faculty of Oral and Dental Medicine, Future University in Egypt, Cairo, 11835 Egypt

**Keywords:** *Acacia nilotica*, *Culex pipiens*, Medicinal plants, Phytochemicals, Molecular simulation, Structure activity relationship, Biological techniques, Zoology, Medical research, Chemistry

## Abstract

Mosquitoes are one of the deadliest and most hazardous animals on Earth, where they transmit several diseases that kill millions of people annually. There is an ongoing search almost everywhere in the world for more effective and contemporary ways to control mosquitoes other than pesticides. Phytochemicals are affordable, biodegradable biological agents that specialize in eliminating pests that represent a risk to public health. The effectiveness of *Acacia nilotica* methanol and aqueous leaf extracts against 4th instar larvae was evaluated. The results revealed that the methanol extract of *A. nilotica* had a noticeable influence on the mortality rate of mosquito larvae, especially at high concentrations. Not only did the mortality rate rise significantly, but the hatching of the mosquito eggs was potentially suppressed.Terpenes, fatty acids, esters, glycosides, pyrrolidine alkane, piperazine, and phenols were the most prevalent components in the methanol extract, while the aqueous extract of *A. nilotica* exclusively showed the presence of fatty acids. The insecticidal susceptibility tests of both aqueous and alcoholic extract of *A. nilotica* confirmed that the *Acacia* plant could serves as a secure and efficient substitute for chemical pesticides because of its promising effect on killing larvae and egg hatching delaying addition to their safety as one of the natural pesticides. Molecular docking study was performed using one of the crucial and life-controlling protein targets, fatty acid binding protein (FABP) and the most active ingredients as testing ligands to describe their binding ability. Most of the structurally related compounds to the co-crystallized ligand, OLA, like hexadecanoic acid furnished high binding affinity to the target protein with very strong and stable intermolecular hydrogen bonding and this is quite similar to OLA itself. Some other structural non-related compounds revealed extraordinarily strong binding abilities like Methoxy phenyl piperazine. Most of the binding reactivities of the majortested structures are due to high structure similarity between the positive control, OLA, and tested compounds. Such structure similarity reinforced with the binding abilities of some detected compounds in the *A. nilotica* extract could present a reasonable interpretation for its insecticidal activity via deactivating the FABP protein. The FABP4 enzyme inhibition activity was assessed for of both methanolic and aqueous of acacia plant extract and the inhibition results of methanol extract depicted noticeable potency if compared to orlistat, with half-maximal inhibitory concentration (IC_50_) of 0.681, and 0.535 µg/ml, respectively.

## Introduction

Mosquitoes are a crucial insect because of their significance as a vector in disease transmission^1^. Diseases including dengue, malaria, filariasis, west nile fever, lumpy skin disease, yellow fever, and Japanese encephalitis can be spread by them^2–6^. In recent years, dengue viruses, which are spread by infected female *Culicidae* mosquitoes, specifically *Aedes aegypti* and *Aedes albopictus*—have grown to be a serious global public health problem^7–9^. Gibbons identified *Aedes aegypti* as the primary vector of arboviral dengue virus infections in tropical and subtropical regions^10,11^. Worldwide, between 50 and 100 million people contract the disease each year, and around 2.5 percent of those cases result in death^12^.

The annual incidence of malaria is approximately 4–5 million in poor countries, such as India, where about 236 million people live in filariasis-endemic areas. Dengue fever still poses a threat to millions of people in different parts of the globe^13^. The most important vectors implicated in disease transmission to humans are the genera *Culex, Aedes,* and *Anopheles*^14^.

Several control approaches are routinely employed to control mosquito-borne risks. Insects are compelled to adapt to synthetic insecticides, which have been around for a while. Moreover, continuous use of pesticides poses concerns to the ecosystem since insects are becoming resistant to the chemicals^15^. Phytochemicals represent a significant substitute for synthetic insecticides. Mosquitoes are affected by phytochemicals in a number of ways, including as oviposition deterrent, developmental toxin, hatching blocker, adulticidal, ovicidal, and emergence blocker^16^.

The effectiveness of several medicinal plant preparations as larvicidal agents or as mosquito repellents has not yet been determined. Recently, plant-based insecticides and those that don’t hurt the environment have received increased attention while creating new pesticides^17–19^. Therefore, natural substances are the ideal alternative since they are eco-friendly, bio-sourced, and safe to use, causing less harm to the ecosystem and non-target species. Furthermore, a variety of extracts and compounds derived from diverse plant families have been investigated as possible new larvicides^20^. Studies have revealed that saponin^21^, steroids^22^, isoflavonoids^23^, essential oils^24^, alkaloids^25^, and tannins^26^ are all effective against mosquito larvae.

The plants are known to have an extensive number of bioactive chemicals that are both sustainable and regenerative. Royal Poinciana, *Delonix regia* is one of most interested plants which are widely distributed in temperate and tropical regions, and its leaves have long been used in folk medicine, notably as an antimalarial, particularly in East Asia. A wide spectrum of bioactivities was reported for the *Delonix Regia* extract. The plant has demonstrated positive antioxidant and antimicrobial properties^27–29^. Additionally, it has been reported that the flower's acetone and methanol extracts have effective larvicidal properties.

Plant products are one of the finest solutions for mosquito control, and numerous plant treatments were attempted before the discovery of chemical pesticides^30–33^. Moreover, the expanding trend and favorable community reaction to phytochemicals and their ecologically beneficial behavior offer an open field for plant-based pesticide research and innovation. Keeping in view the toxic activities of the family Fabaceae and many other plant families, which were demonstrated against other mosquito species^34,35^.

The Domain of therapeutical targets and drug design has been developed and expanded after the full accomplishment of human genome project, synchronizing with extensive and devolving advanced strategies throughputs related to x-rays diffraction (XRD), nuclear magnetic resonance (NMR) and Fourier transform infra-red (FTIR) and other structure- identifying tools permits to more success in knowing both protein–ligand and protein complex structure easily^36^. The rapid and effectual in structure identifications lead to a jump in computer-aided drug design and consequently the molecular docking, which presented a theoretical-based simulation between the drug and the host protein to a special part called pocket, such interactions are controlled by some sophisticated calculations. Nowadays, most of the research articles comprise one of the artificial intelligence applications especially that deals with ligand–protein^37^ and protein–protein interactions^37^. Synthetic and therapeutical chemistry are one of the most probable branches that heavily depends on the drug design and molecular docking^38^. The molecular simulation docking facilitates the selection of the most powerful candidate form huge databases and consequently facilitating the Structure Activity Relationship (Q-SAR) which connect between the structure and its biological impact. Such theoretical studies linked the drug candidate physical activity, e.g., biological activity, and their structure, making the biological activity easier to be predicted before the chemical synthesis^39^. Molecular docking used in various chemical design and synthesis of some EGFR analogues^40^, PI3k^41^, carbonic anhydrase^42^, acetylcholinesterase^43^, topoisomerase^44^ and m-TOR inhibitors^45^.

The effectiveness of plant extracts and its activated ingredients against mosquito larvae varies greatly depending on many factors related to the plant itself environment that the plant grew up, components of the plant used, its age (young, mature, or senescent), and the solvent used during the extraction. So, this study was designed to compare the lethal effects of some Fabaceae plants on filarial vectors *Cx. pipiens* and Rift Valley fever, *Ae. aegypti*. Moreover, both aqueous and methanolic acacia extract were prepared and the most activated ingredients were expected using GC/MS analysis and some of the detected compounds at high ratio were taken into consideration. All detected compounds were investigated to the Molecular docking simulation to assess their activity to interact and inhibit FABP protein, where 2flj, fatty acid binding protein, was used as the target protein and the most abundant active ingredients in the plant extract as tested ligands. The structure activity relationship between OLA and most of the tested compounds helps to give a convenient interpretation of the inhibition abilities to the FABP protein and may interpret the potential biochemical change due to the treatment using both types of acacia extracts.

## Materials and methods

### Ethical statement

All plant experiments in the study were conducted in accordance with relevant guidelines and legislation of ethical committee of Benha University.

### Plant materials and analysis

#### Plant collection and extraction

Leaves of Acacia plants, *Acacia nilotica* (Fabaceae) were collected from different areas in Qalyubiya Governorate, Egypt, during August–September 2022. Plants were identified in the Flora and Phytotaxonomic Section of the Agricultural Research Centre in Giza, Egypt. The plant components were shade-air-dried at room temperature for seven days until the plant was thoroughly dried and the dry weight contracted. To keep the dried tissues free from moisture, they were pulverized in an electric mixer made of stainless steel and placed within airtight containers. To make the methanol extract, 20 g of dry plant materials were soaked in 100–150 ml of methanol in a conical flask (cap. 250 ml) at room temperature 27 ± 2 °C. Another 20 g of plant components were mixed with warm water (60 °C) at room temperature. After 48 h, the solutions were filtered via a Buchner funnel using Whatman No. 1 filter paper. A rotary evaporator was used to concentrate the extract for 3–5 h at room temperature (28 °C) and solid product then stored at 4 °C until used^46^.

### Mosquito larvicidal assay

#### *Culex pipiens* rearing

Larvae of *Cx. pipiens* were raised in the insectary at the Department of Entomology, Faculty of Science, Benha University, Egypt, for 12 generations, where they were kept at 27 ± 2 °C and 75 ± 5% relative humidity during a light/dark photoperiod of 12:12 h. The larvae were fed a 3:1 ratio of ground bread to fish meal (Tetramin). After being transferred from the enamel pans to a cup of dechlorinated water, the pupae were placed in screened cages of 35 × 35x40 cm, from which the adults finally emerged. The adult mosquito colony received a 10% sugar solution, while the females were periodically fed blood by a hamster rat. Two developmental stages, larvae, and pupae were consistently accessible for the testing and maintained in the same lab.

#### Larvicidal activity

Under laboratory circumstances, the plant leaf methanol, and aqueous extracts of *A. nilotica* were tested against the 4th larval instar of *Cx. pipiens* (No. = 20 larvae). The fourth larval instar was exposed to doses of 62.5, 125, 250, 500, 1000, and 1500 ppm (1 g/1000 ml distilled water). Twenty larvae were introduced to a glass beaker holding 250 ml of distilled water for each concentration. Each concentration received three replicates. Temephos was employed at a rate of 0.25 ppm as a positive control according to WHO^47^ standard concentration within a similar volume of test beaker^48^. Mortality rates were obtained 24 h, 48 h, and 72 h after the first exposure and post-treatments (PT)^12^. No food was provided to the larvae during the experimental period.

### Oviposition assay

To analyze the effects of *Acacia* extract concentration on oviposition, three concentrations of 250, 500, and 1000 ppm in transparent glass beakers (6.5 cm high and 4.5 cm diameter, volume 200 ml) were prepared and exactly, 75 *Cx. pipiens* females were tested. After taking the blood meal, the number of egg rafts in the three cups was monitored for four to five days. The experiment was repeated three times with untreated control groups (dechlorinated tap water only). The hatching rate of mosquito eggs was also determined according to Zheng et al.^49^, where all egg rafts were examined under binocular microscope**.** Hatch rate was calculated after 72 h by dividing the number of eggs observed and considered to be hatched by the total number of eggs present on the paper.

### Efficacy of selected plant extracts against non-target predators

*Acacia* methanol and aqueous extracts were tested against *Gambusia affinis* and *Sphaerodema urinator* and, two of the among field predators that live in mosquito’s places. After catching, they were placed in plastic bags partially filled with water and transported to the lab. 24 h, predators were fasted prior to the bioassay. They were then treated with LC_50_ of Acacia extract, 3 predators per 1 l of dechlorinated water inside an enamel plate (35 × 25 × 15 cm) with 300 larvae. Three replicates were carried with controls.

### GC/MS analysis

For the biochemical analyses, Thermo Scientific Trace GC Ultra/ISQ Single Quadrupole MS, TG-5MS fused silica capillary columns were utilized, with thicknesses of 0.1 mm, 0.251 mm, and 30 m. It was done with a 70 eV ionization energy electronic ionizer. Helium gas was employed as a carrier gas (flow rate: 1 ml/min). The MS transmission line and injector were both set to 280 °C. Preheating the oven to 35 °C, it was then raised to 150 °C at a rate of 7 °C per minute, 270 °C at a rate of 5 °C per minute (with a two-minute break in between), and finally 310 °C at a rate of 3.5 °C per minute (continual for 10 min). To investigate the quantification of every component found, a relative peak area was utilized. The substances were at least partially identified by matching their retention periods and mass spectra to NIST and Willy Library data from the GC/MS instrument. The aggregate spectrum of user-generated reference libraries was used for identification. Single-ion chromatographic reconstructions were carried out to assess peak uniformity. When possible, co-chromatographic analysis of reference chemicals was employed to validate GC retention durations^50^.

### Fatty acid binding protein inhibition assay

Th inhibition assay of Fatty acid binding protein (FABP4) was determined according to Zimmerman and Veerkamp^51^. The solid aqueous and methanolic extracts were dissolved in DMSO to make the required serial dilution which has no detrimental effect on the fatty acid binding protein. FABP assay buffer was prepared via dilution of the (10 × buffer) by taking 2 ml and 18 ml HPLC-grade water. Followed by preparation of FABP assay detection reagent, FABP4 assay protein, arachidonic acid and inhibitor/ligand concentrations according to Cayman chemical kit recommendations. In 96 well plat, 40 µl of FABP assay buffer to each well followed by adding of 25 µl of the detection reagent and 10 µl of the inhibitor/ligand dilution. Then the plates were covered and incubated for 10 min and the fluorescence measured at 475 nm after excitation at 370 nm using a plate reader.

### Molecular docking study

#### Source of the objective protein

The binding affinity of the recognized active ingredients from both aqueous and alcoholic acacia extract against *lm*-FABP binding site were examined using one of the theoretical approaches in molecular docking, MOE tool, to decide whatever the active ingredients could make successive binding with the target protein and accordingly suggesting the mode of action. There is not a three-dimensional structure of fatty acid binding protein owing to *Cx. pipiens* in the protein data bank, hence the well-known crystal three-dimensional structure of the Fatty acid binding protein from locust flight muscle in complex with oleate *lm*-FABP(PDB code:2FLJ)^43^ was utilized for this purpose and downloaded from protein data bank (https://www.rcsb.org/structure/2FLJ) as PDB format, water molecules were excluded in addition to any heteroatom subtracted and only chain a constrained.

#### Energy minimization

The most abundant and activated ingredients from both aqueous and alcoholic acacia extracts were automatically identified by the GC/MS. A database set of twenty candidates of the active ingredients were chosen to perform this study, were drawn using CAMBRIDGESOFT CHEMOFFICE 2015 Professional 15.0.0 software after recalling their smiles from PubChem database. The drawn structures were saved as Mol format after fulfillment energy minimization step using the defaults’ function Amber12: EHT forcefield till gradient convergence of 0.01 kcal/mol. Energy minimization step in addition to molecular simulation were done using the software Molecular Operating Environment MOE_2015.10, which installed by 64-bit operating system [Intel (R) Core (TM) i5-2400 CPU @ 2.40 GHz, 8 GB RAM] system.

#### Docking procedure

After uploading *lm*-FABP (PDB code: 2FLJ), both twenty candidate ligands and target protein were prepared, the reference drug (oleate) was assorted in green color to be distinguished easily. The binding site was recognized automatically from the surfaces and maps option and accordingly the co-crystallized ligand’s binding site, pocket site, was separated and saved as MDB file. After assorting the twenty candidates in energy-minimized in new database, the docking step was done from “compute” option using the defaults of “Rotate bonds” choice to permits flexible-ligand rigid receptor docking to be fulfilled. Additionally, the scoring energy function was changed to be London G with triangle matcher replacement were set. A “fifteen conformers” was re-adjusted instead of the “thirty” automatic option conformers of the best score energy done by the ligand. One of top five conformers of best energetically favored ligand–protein docking was chosen to represent the ligand docking and showed two-and three-dimensional receptor interactions^52^. Docking results of all the tested twenty candidates were assorted into one table, considering the preference of number of interactions, scoring energy (kcal/mol), RMSD (Å), and the bond length (Å). Three- and two-dimensional docking interaction were captured, and the green color was used to indicate the ligand under docking investigation to be easily distinguished, intermolecular hydrogen bonding and π-π staking (aromatic) were labelled in magenta and yellow color, respectively, meanwhile the loops, helical, etc. of protein structures were colored automatically by the software and some renderings set up are made for best manifestation show.

### Fatty acid binding protein activity test (FABP4)

Fatty acid binding protein (FABPs) are group of intracellular liquid binding protein (iLBP) usually binds to intracellular hydrophobic ligands and shielding them throughout cellular compartments. The activity of FABP4 caused by both aqueous and methanolic acacia extracts treatment were observed to be diminished and the minimal inhibitory concentration caused by methanolic extract 0.681 µg/ml meanwhile aqueous extract 2.311 µg/ml against the positive control, orlistat, 0.535 µg/ml. Studies conducted in FABP4 levels in mice have proven that the down-fluctuations of such proteins are related to many metabolic diseases^53,54^. With crucial role future clinical treatments. Various effective FABP4 inhibitors were developed but unfortunately, none of them approved or even entered clinical trials^55^. BMS-309403, FABP4-ap2, is a very strong, selective adipocyte fatty acid binding protein inhibitor with minimal inhibitory concentration *ki* = 2 nM (0.9 µg/l), 250(118 µg/l), and 350(165 µg/l) nM for FABP4, FABP3, and FABP5, respectively^56^. Comparatively, Methanolic acacia extract exhibited good inhibition ability with *ki* = 0.68 mg/l and being from natural source that may encourage its safety attitude.

### Statistical analysis

The data were analyzed using the software SPSS V23 (IBM, USA) to perform Probit analyses to compute lethal concentration (LC) values and the one-way analysis of variance (ANOVA) (Post Hoc/Turkey's HSD test). The significance threshold was established at *P* < 0.05.

## Results

### Mosquito larvicidal activity

This study assessed *A. nilotica* plant extracts on *Cx. pipiens* larvae in their fourth instar. In this study, every tested plant extract shown strong larvicidal efficacy against *Cx. pipiens* mosquito larvae. The findings of this study demonstrated that methanol extracts of plant materials had stronger harmful effects than aqueous extracts against *Cx. pipiens* mosquito larvae.

The mortality percent (MO%) of *Cx. pipiens* treated with 1500 ppm methanol extracts of *A. nilotica* at 24 h post-treatment (PT) was 100% (Table 1) and the corresponding LC_50_ (50%, median lethal concentration) = 200.50 ppm (Table 2), while the corresponding values for aqueous extracts were 98 (MO%) and the LC_50_ values = 256.40 ppm.

**Table 1:** Efficacy of *Acacia nilotica* methanol and aqueous extracts on *Cx. pipiens* mortality, 24 and 48 h post-treatment.

Solvent	Conc. (ppm)	*Acacia nilotica*	
		24 h	48 h
Aqueous	Control	0 ± 0^gA^	0 ± 0^fA^
	62.5	11 ± 1.00^fB^	15 ± 1.58^eA^
	125	20 ± 1.58^eB^	34 ± 2.92^dA^
	250	45 ± 2.24^dB^	65 ± 2.24^cA^
	500	70 ± 2.24^cB^	85 ± 4.47^bA^
	1000	85 ± 2.24^bB^	96 ± 0.00^aA^
	1500	98 ± 2.00^aA^	100 ± 0.00^aA^
Methanol	Control	0 ± 0^gA^	0 ± 0^fA^
	62.5	15 ± 2.24^fB^	19 ± 2.92^eA^
	125	25 ± 2.24^eB^	39 ± 1.87^dA^
	250	59 ± 4.30^dB^	71 ± 2.92^cA^
	500	80 ± 4.47^cB^	96 ± 2.45^bA^
	1000	95 ± 3.16^bB^	100 ± 0.00^aA^
	1500	100 ± 0.00^aA^	100 ± 0.00^aA^
Temephos*	0.25	73.3 ± 4.47^cB^	100 ± 0.00^aA^

Numbers of the same column followed by the same small letter (a–g) are not significantly (*P* > 0.05) different (one-way ANOVA, Duncan’s MRT). * Temephos insecticide at 0.25 ppm.

**Table 2:** Lethal concentrations (ppm) of *Acacia nilotica* methanol and aqueous extracts against *Cx. pipiens*, 24 and 48 h post-treatment.

Solvent	Time (h)	LC_50_ (Low-Up.)	LC_90_ (Low-Up.)	LC_95_ (Low-Up.)	Slope ± SE	Chi (Sig.)
Aqueous	24	256.40 (192.44–335.07)	847.23 (657.93–1328.31)	1188.91 (909.91–2010.86)	2.469 ± 0.162	9.944 (0.041)
	48	172.68 (152.46–194.28)	531.34 (452.54–647.40)	730.70 (604.27–928.39)	2.6256 ± 0.1851	4.898 (0.297)
Methanol	24	200.50 (181.02–232.02)	711.24 (615.99–870.81)	1029.21 (854.02–1293.05)	2.352 ± 0.174	3.863 (0.424)
	48	140.13 (127.97–161.59)	408.42 (353.90–485.95)	548.38 (463.19–676.85)	2.8389 ± 0.1971	3.565 (0.468)

The larval mortality was maximum after 48 h of PT, while the mortality reaching 100% at 1500 ppm in methanol and aqueous extracts. In terms of fatal concentrations, *A. nilotica* methanol extracts revealed to be the most effective against *Cx. pipiens* larvae (LC_50_ = 140.13 ppm), followed by aqueous extracts (LC_50_ = 172.68 ppm). The larval mortality rate for the traditional positive control (temephos) was 73.3 and 100% at 24 and 48 h post treatment, respectively in aqueous and Methanol extracts.

### Effect of *Acacia* extract concentration on eggs hatching rate

**Figure 1:**
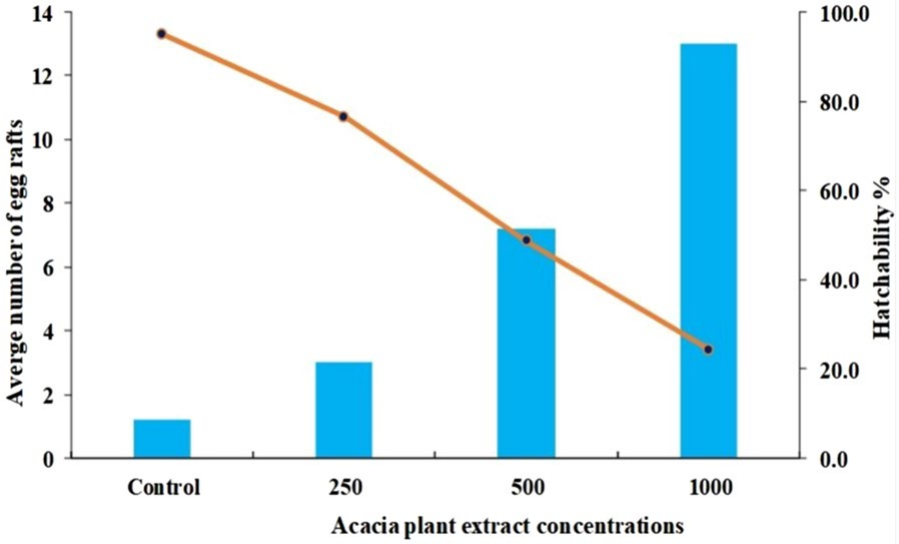
Efficacy of *A. nilotica* plant extracts concentration on the oviposition and hatching rates of female *Cx. pipiens.*

We tested the oviposition and hatchability rate of eggs in the *A. nilotica* solution. Exactly 25 *Cx. pipiens* females were used for the test and mixed with 30 of the mosquito males, and consequently, 122 egg rafts were counted, and the detailed number of laid eggs and hatching rate were summarized in Table 3 and Fig. 1. At a higher concentration of *A. nilotica* extract (1000 ppm), about 65 egg rafts were laid and reached a hatching rate of 24.4%. At the moderate concentrations (500 ppm), a moderate number of egg rafts were laid (37 eggs) with a 48.4% hatching rate compared to the low-concentrated *A. nilotica* extract (250 ppm), which produced around 15 egg rafts with a hatching rate of 76.5% of the total eggs laid. The t-test demonstrated that these results were statistically significant (*P* < 0.05).

**Table 3:** Efficacy of plant extracts concentration on oviposition rates of female *Culex pipiens*.

Conc. (ppm)	Mean number of eggs rafts oviposited ± SD	*P* value	Number of eggs laid/female	Hatching %
0	1.20 ± 0.4*	0.000	6.2	95
250	3.00 ± 0.4*	0.000	15.6	76.5
500	7.20 ± 0.9*	0.000	37.4	48.8
1000	13.00 ± 2.3*	0.001	67.6	24.4

*The results showed no significant difference (*P* > 0.05).

### The Efficacy of plant extracts against non-target predators

The effects of the *Acacia* plant extracts on non-target insects (G*. affinis* and *S. urinator*) were tested. They were treated with LC_90_ values of *A. nilotica* aqueous (847.23), and *A. nilotica methanol* (711.24 ppm), respectively, against these predators (Table 4). The mean predation for *A. nilotica* methanol and aqueous with chosen predators did not differ significantly (*P* > 0.05). According to the data, mosquito fish, *G. affinis* (78.67, 80.67%) and *S. urinator* 26, 25.67%) bug were not affected in both methanol and aqueous plant extracts (Table 4).

**Table 4:** The mean number of mosquito larvae, *Culex pipiens* consumed by *Gambusia affinis* and *Sphaerodema urinator* predators before and after treated with *Acacia* extracts under laboratory conditions.

Mosquito predator	Plant extract types	
	Aqueous	Methanol
Control	89.67 ± 0.33^aA^	89.67 ± 0.33^aA^
*G. affinis*	80.67 ± 1.33^bA^	78.67 ± 0.67^bB^
Control	29.00 ± 0.58^cA^	29.00 ± 0.58^cA^
*S. urinator*	25.67 ± 0.67^dA^	26.00 ± 0.58^dA^

a, b and c: There is no significant difference (*P* > 0.05) between any two means, within the same column that have the same superscript letter. A, B and C: There is no significant difference (*P* > 0.05) between any two means for the same attribute, within the same row have the same superscript letter.

### Phytochemical analysis

**Figure 2:**
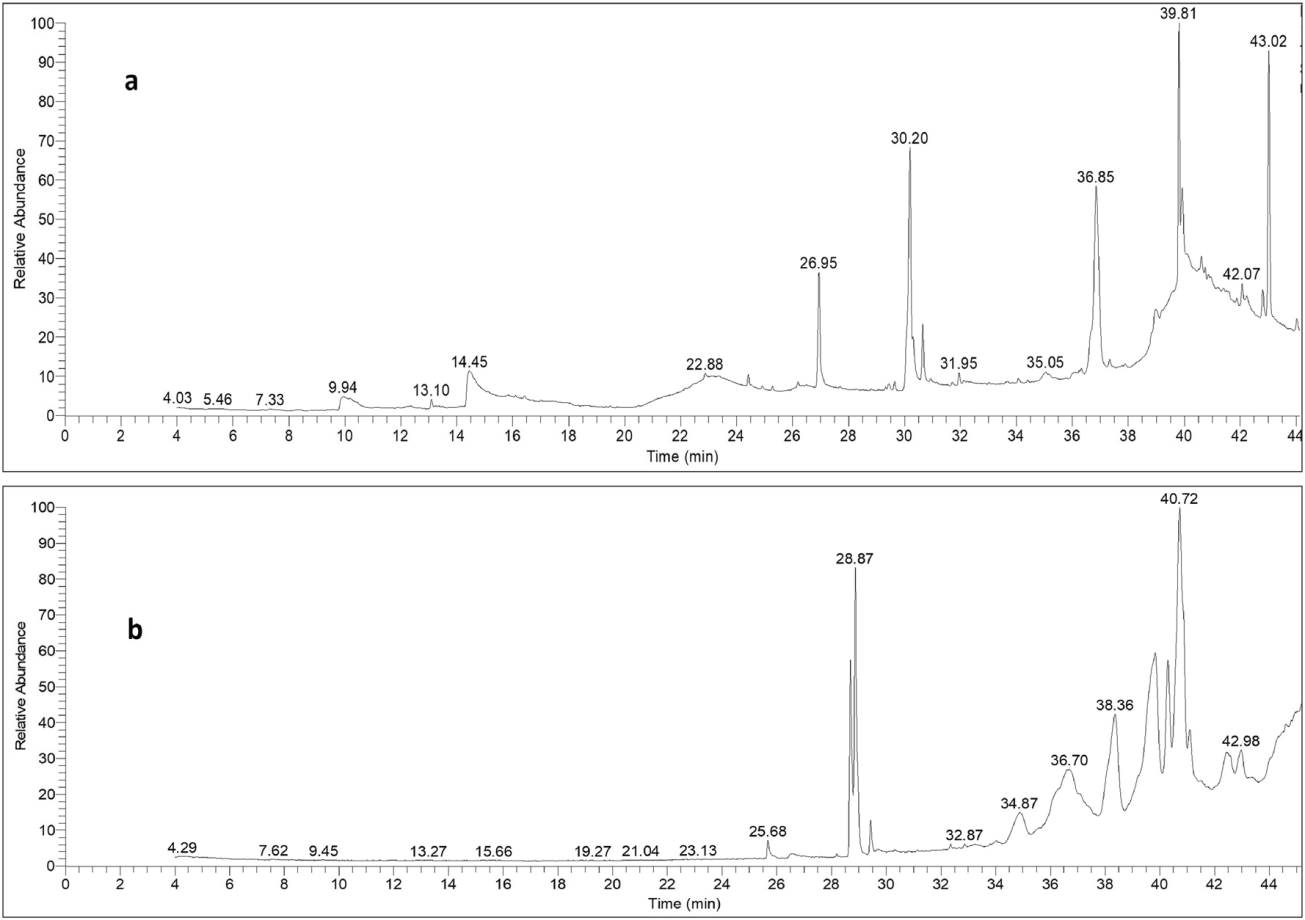
The TIC chromatograms of *Acacia nilotica* methanol (**a**) and aqueous extracts (**b**) chemical constituents detected by GC–MS.

GC–MS analysis was used to perform a metabolic investigation of *A. nilotica* extracts and a comparison of aqueous and methanol extracts. Our study’s GC–MS analysis results allowed us to identify a variety of chemicals in the leaves of *A. nilotica*, including phenols, terpenes, esters, glycosides, fatty acids, piperazine, pyrrolidine and alkane (Tables 5 and 6). Methanolic leaf extract of *A. nilotica* showed the presence of 19 compounds (Table 5 and Fig. 2a), while aqueous leaf extract of *A. nilotica* showed 6 compounds (Table 6, Fig. 2b). Among them, 9,12,15-Octadecatrienoic acid (21.10%), squalene (18.25%), stigmasterol (14.25%), and 2,3-dimethoxy-anthracene-9,10-dione (5.65%) showed abundance in methanolic extract while, Cis-11-eicosenoic acid (68.10%), 11-Octadecenoic acid, methyl ester (20.14%), and 9,12-Octadecadienoic acid (z,z)-, methyl ester (8.80%) showed abundance in aqueous extract.

**Table 5:** The major chemical constituents of *Acacia nilotica* methanol extracts.

No	RT	Area %	Compound Name	M. F	M.W
			*Piperazine*		
1	16.42	0.64	Methoxyphenyl piperazine	C_11_H_16_N_2_OS	224
			*Cyclohexane*		
			*Faty acids and esters*		
2	24.42	1.74	12-tridecynoic acid, methyl ester	C_14_H_24_O_2_	224
3	26.20	0.35	Pentadecanoic acid, 14-methyl-, methyl ester	C_17_H_34_O_2_	270
4	26.95	5.00	Hexadecanoic acid	C_16_H_32_O_2_	256
5	29.65	1.95	Methyl z-11-tetradecenoate	C_15_H_28_O_2_	240
6	30.20	21.10	9,12,15-Octadecatrienoic acid	C_18_H_30_O_2_	278
7	36.85	14.25	Stigmasterol	C_29_H_48_O	412
8	38.81	2.24	7,10,13-eicosatrienoic acid, methyl ester	C_21_H_36_O_2_	320
			*Terpene (Monoterpene and Sesquiterpene)*		
9	39.81	18.25	Squalene	C_30_H_50_	410
			*Pyrrolidine alkaloid*		
10	36.33	1.47	1,8-diethyl-3,6-diazahomoadamantan-9-ol	C_13_H_24_N_2_O	224
			*Phenols*		
11	9.88	1.41	Brenzkatechin	C_6_H_6_O_2_	110
12	14.41	2.99	1,2,3-benzenetriol	C_6_H_6_O_3_	126
13	37.33	5.65	2,3-dimethoxy-anthracene-9,10-dione	C_16_H_12_O_4_	268
			*Steroid andPolyethylene glycol*		
14	42.07	3.31	9,10-secocholesta-5,7,10(19)-triene-3,24,25-triol, (3á,5z,7e)	C_27_H_44_O_3_	416
			*Glycoside*		
15	42.23	1.78	4 h-1-benzopyran-4-one,2-(3,4-dihydroxyphenyl)-6,8-di-á-d-glucopyranosyl-5,7-dihydroxy-	C_27_H_30_O_16_	610

**Table 6:** The major chemical constituents of *Acacia nilotica* aqueous extracts.

No	RT	Area %	Compound Name	M. F	M.W
			*Faty acids and esters*		
1	25.68	1.01	Hexadecanoic acid, methyl ester	C_17_H_34_O_2_	270
2	28.69	8.80	9,12-Octadecadienoic acid (z,z)-, methyl ester	C_19_H_34_O_2_	294
3	28.87	20.14	11-Octadecenoic acid, methyl ester	C_19_H_36_O_2_	296
4	29.43	1.11	Stearic acid methyl ester	C_19_H_38_O_2_	298
5	40.72	68.10	Cis-11-eicosenoic acid	C_20_H_38_O_2_	310
6	44.28	0.84	Ethyl iso-allocholate	C_26_H_44_O_5_	436

### FABP4 enzyme assay evaluation

**Figure 3:**
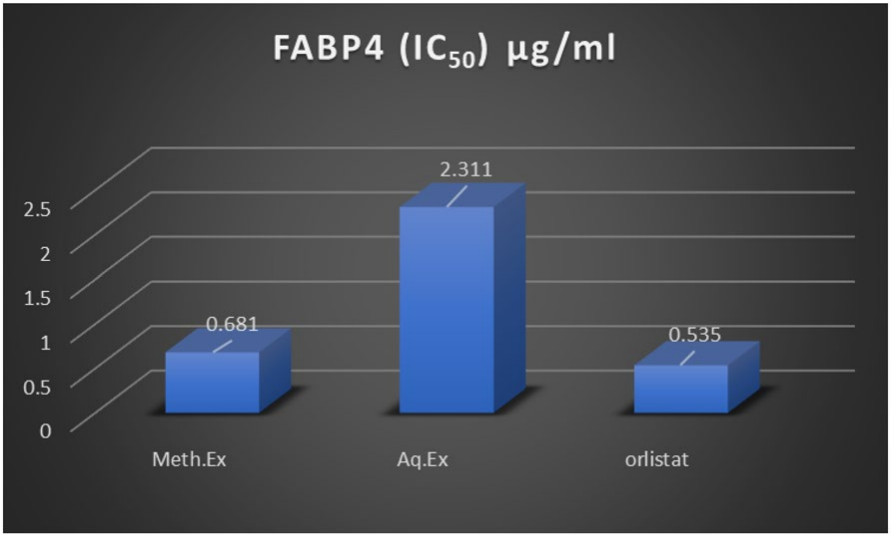
FABP4 enzyme assay inhibition of the tested acacia extracts.

The FABP4 enzyme inhibition assay for both methanolic and aqueous of acacia plant extract were estimated and orlistat was used as positive control and their half-maximal inhibitory concentration (IC_50_) 0.681, 2.311 and 0.535 µg/ml for the methanolic extract, aqueous extract and the lipase inhibitor, orlistat, respectively (Fig. 3).

### Docking study

**Figure 4:**
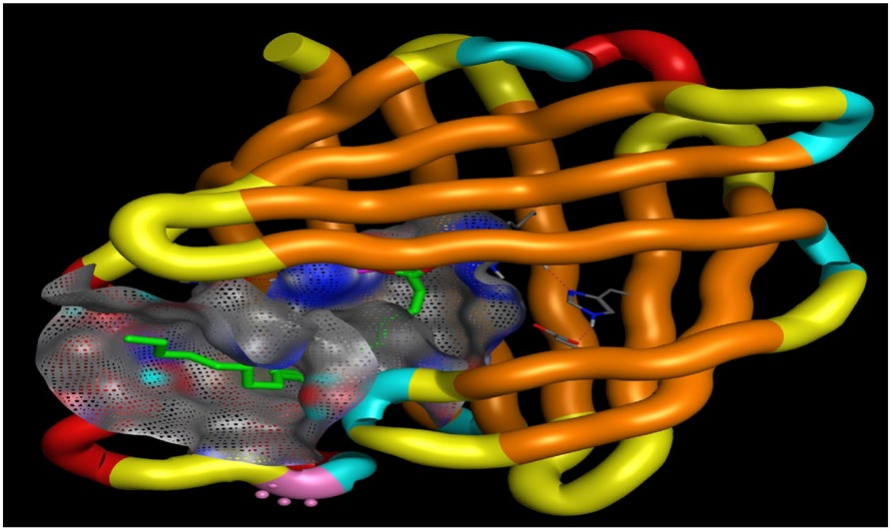
Three-dimensional structure of 2flj and its active pocket.

**Figure 5:**
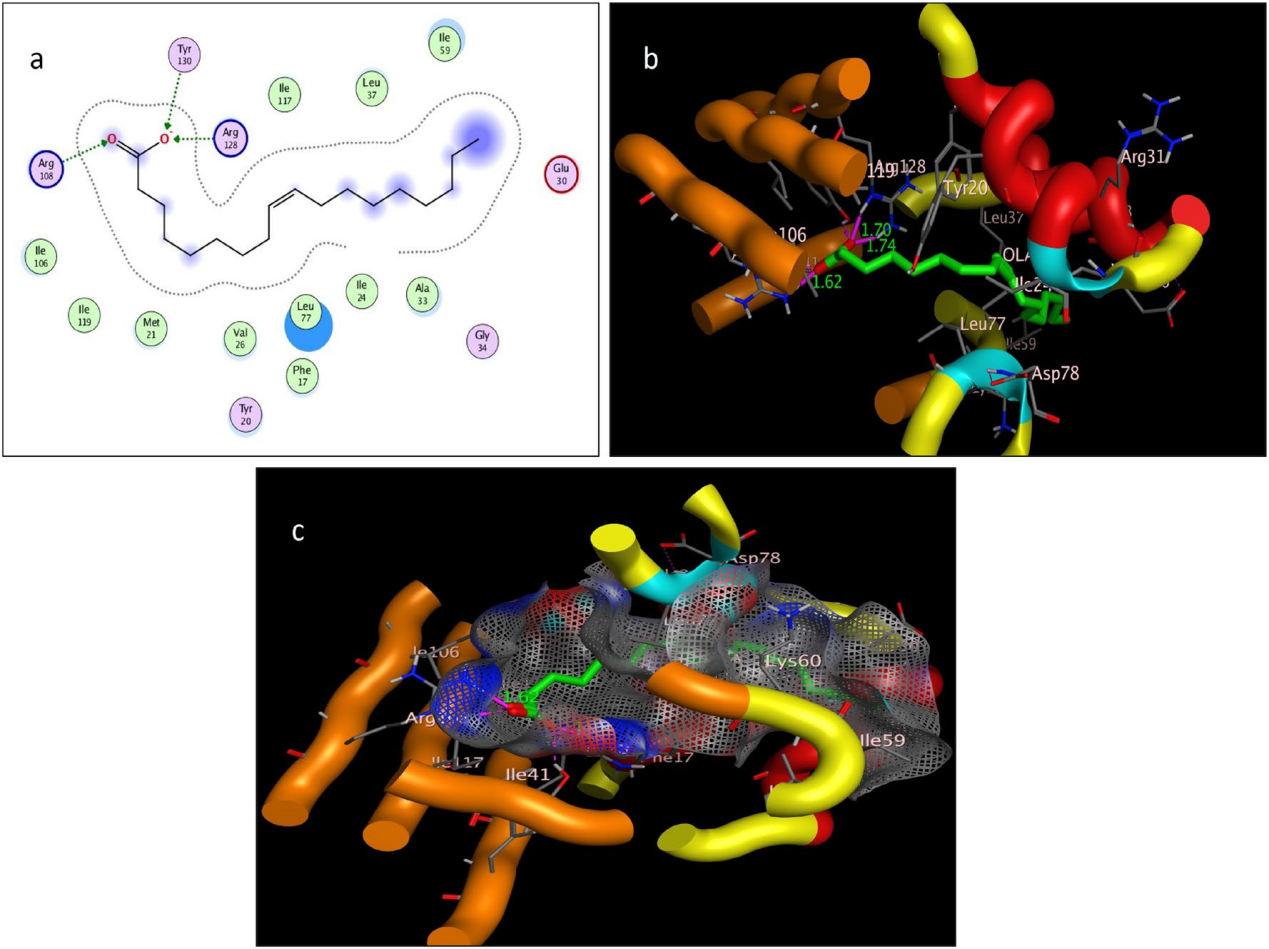
Self-Docking of the co-crystallized ligand (Oleic acid or OLA) interior 2FLJ pocket: (**a**) two-dimensional receptor interactions; (**b**) three-dimensional receptor interactions; (**c**) three-dimensional positioning in the receptor pocket.

**Figure 6:**
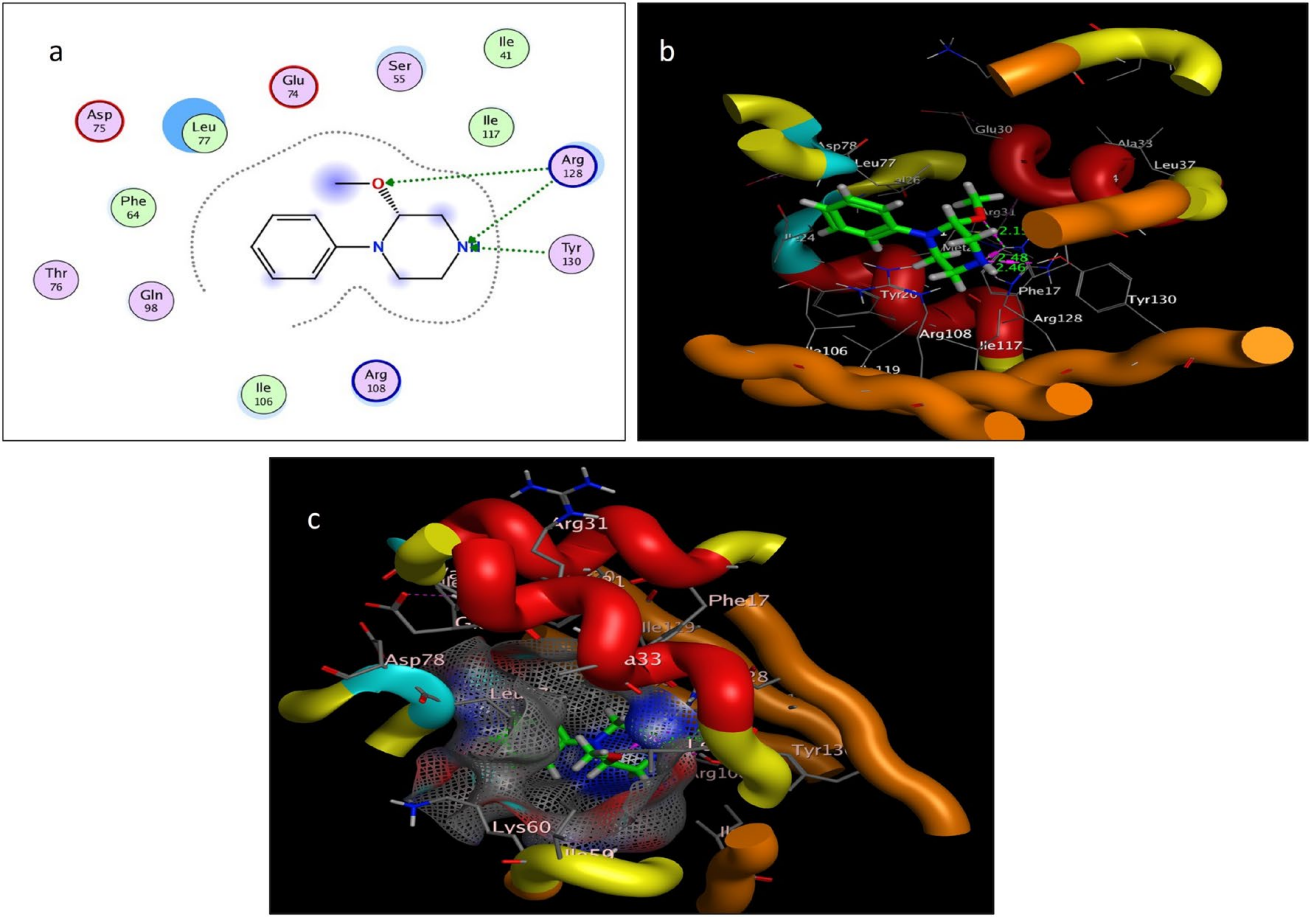
Ligand-Docking of compound 1 (Methoxy phenylpiperazine) interior 2FLJ pocket: (**a**) two-dimensional receptor interactions; (**b**) three-dimensional receptor interactions; (**c**) three-dimensional positioning in the receptor pocket.

**Figure 7:**
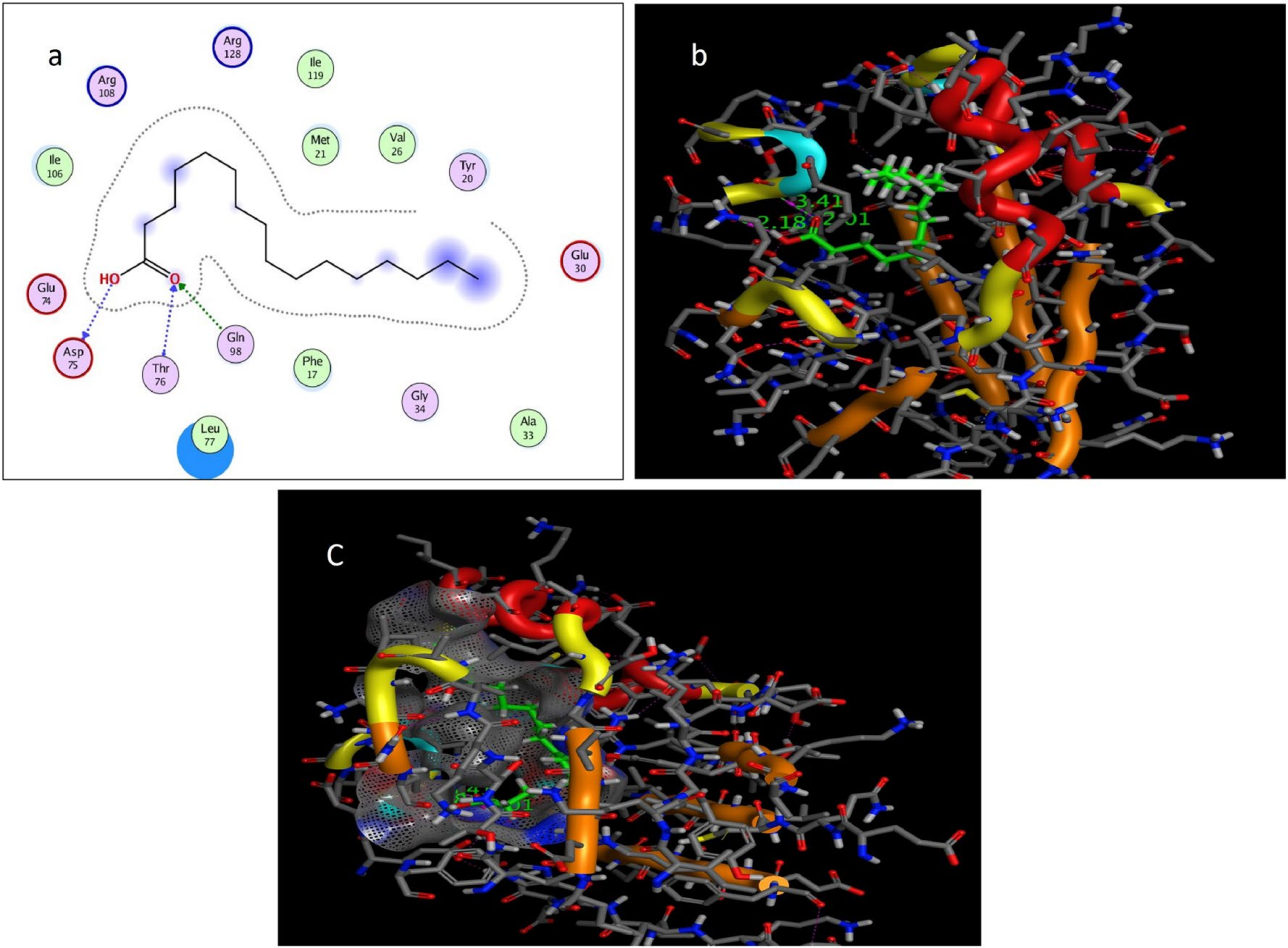
Ligand-Docking of compound 4 (hexadecanoic acid) interior 2FLJ pocket: (**a**) two-dimensional receptor interactions; (**b**) three-dimensional receptor interactions; c) three-dimensional positioning in the receptor pocket.

The target protein *Lm*-FABP, PDB:2FLJ as fatty acid binding protein in Fig. 4 was chosen to perform docking study of twenty candidates of the most abundant active ingredient in aqueous and methanolic acacia extracts included: of Methoxyphenyl piperazine **[1]**, 12-tridecynoic acid, methyl ester **[2]**, pentadecanoic acid, 14-methyl-, methyl ester **[3]**, hexadecanoic acid **[4]**, methyl z-11-tetradecenoate **[5]**, (z,z,z)-9,12,15-octadecatrienoic acid **[6]**, stigmasterol **[7]**, 7,10,13-eicosatrienoic acid, methyl ester **[8]**, squalene **[9]**, 1,8-diethyl-3,6-diazahomoadamantan-9-ol **[10]** Brenzkatechin **[11]**, 2,3-dimethoxyanthracene-9,10-dione **[12]**, (3á,5z,7e) 9,10-secocholesta-5,7,10(19)-triene-3,24,25-triol **[13]**, Hexadecanoic acid, methyl ester **[14]**, 11-Octadecenoic acid, methyl ester **[15]**, 9,12-Octadecadienoic acid (z,z)-, methyl ester **[16]**, Stearic acid methyl ester **[17]**, Cis-11-eicosenoic acid **[18]**, Ethyl iso-allocholate **[19]**, 1,2,3-benzenetriol **[20]** were docked to the active center in the target protein presented in Fig. 4. The final simulation outcomes were listed in Table 7. The Candidate Methoxy phenyl piperazine **[1]** reacted similarly like the co-crystallized ligand, OLA presented in (Fig. 5, and revealed three different dipole–dipole interactions with the residues arginine and tyrosine with bond lengths between 2.15 and 2.48 Å as shown in Fig. 6. Compound **[4]**, hexadecanoic acid, furnished three hydrogen bonds Fig. 7 with Gln98, thr76 and Asp75, at bond length between 2.01 to 3.41 Å. All Compounds (z,z,z)-9,12,15-octadecatrienoic acid **[6]**, 10,13-eicosatrienoic acid, methyl ester **[8]**, 9,12-Octadecadienoic acid (z,z)-, methyl ester **[16]**, Stearic acid methyl ester **[17]**, Cis-11-eicosenoic acid **[18]** and Ethyl iso-allocholate **[19]** shared two intermolecular hydrogen interactions with different residues including arginine and tyrosine as showen in Figs. S1 and S2. Some compounds like 12-tridecynoic acid, methyl ester **[2]**, 1,8-diethyl-3,6-diazahomoadamantan-9-ol **[10]**, Brenzkatechin **[11]**, of 2,3-dimethoxyanthracene-9,10-dione **[12]**, (3á,5z,7e)9,10-secocholesta-5,7,10(19)-triene-3,24,25-triol **[13]** 11-Octadecenoic acid, methyl ester **[15]** and 1,2,3-benzenetriol **[20]** shared less number of hydrogen bonds, only one electrostatic force, as shown in Figs. S3 and S4. Some other compounds have no compatibility to bind to this pocket like pentadecanoic acid, 14-methyl-, methyl ester **[3]**, stigmasterol **[7]** squalene, and Hexadecanoic acid, methyl ester **[14]** as showen in Fig. S5 (Fig. 7).

**Table 7:** Docking results of the most abundant active ingredients in acacia aqueous and methanol extract to the vicinity of *Lm*-FABP, PDB:2FLJ fatty acid binding protein.

Name	No Inter	Residue	Type	Distance (Å)	Score (kcal/mol)	RMSD (Å)
Control (ola)	3	Arg128 → (^–^OC = O)	H-bonding	1.70	–	–
		Arg108 → (O = CO^-^)	H-bonding	1.74		
		Try130 → (^–^OC = O)	H-bonding	1.62		
(1) Methoxy phenylpiperazine	2	Try130 → (N piperazine)	H-bonding	2.15	− 5.0044	0.9219
		Arg128 → (N piperazine)	H-bonding	2.46		
		Arg128 → (OMe exocyclic)	H-bonding	2.48		
(2) 12-tridecynoic acid, methyl ester	1	Gln98 → (O = COMe)	H-bonding	2.02	-6.1885	0.9337
(3) pentadecanoic acid, 14-methyl-, methyl ester	0	No Interactions	–	–	–	–
(4) hexadecanoic acid	3	Gln98 → (O = C–OH)	H-bonding	2.01	− 6.3313	1.3981
		Thr76 → (O = C–OH)	H-bonding	3.41		
		Asp75 → (HO-C = O)	H-bonding	2.18		
(5) methyl z-11-tetradecenoate	1	Leu77 → (O = COMe)	H-bonding	2.01	− 6.3879	1.0141
(6) (z,z,z)-9,12,15-octadecatrienoic acid,	2	His102 → (HO-C = O)	H-bonding	2.42	− 6.7140	1.42301
		Arg80 → (O = C–OH)	H-bonding	2.32		
(7) Stigmasterol	0	No Interactions	–		–	–
(8) 7,10,13-eicosatrienoic acid, methyl ester	2	Try130 → (O = COMe)	H-bonding	2.14	− 7.4504	1.5868
		Arg128 → (O = COMe)	H-bonding	2.04		
(9) squalene	0	No interactions	–		–	–
(10) 1,8-diethyl-3,6-diazahomoadamantan-9-ol	1	Arg108 → (OH)	H-bonding	1.89	− 4.4106	0.4232
(11) brenzkatechin	1	Arg128 → (OH)	H-bonding	2.08	− 4.1880	1.3450
(12) 2,3-dimethoxyanthracene-9,10-dione	1	Arg128 → O = C, anthracene ring	H-bonding	1.95	− 5.8681	0.9982
(13) 9,10-secocholesta-5,7,10(19)-triene-3,24,25-triol, (3á,5z,7e)	1	Arg128 → (OH)	H-bonding		− 4.5683	1.2753
(14) Hexadecanoic acid, methyl ester	0	No interactions	–	–	–	–
(15) 9,12-Octadecadienoic acid (z,z)-, methyl ester	1	Arg108 → (O = COMe)	H-bonding	2.34	− 6.9236	1.8883
(16) 11-Octadecenoic acid, methyl ester	2	Arg108 → OMeC = O	H-bonding	2.12	− 6.6890	1.5963
		Arg128 → (O = COMe)	H-bonding	2.04		
(17) Stearic acid methyl ester	2	Leu77 → (O = COMe)	H-bonding	2.15	− 7.3073	1.7358
		Gln98 → (O = COMe)	H-bonding	2.11		
(18) Cis-11-eicosenoic acid	2	Try130 → (O = C–OH)	H-bonding	2.03	− 6.7317	1.8669
		Arg128 → (O = C–OH)	H-bonding	2.20		
(19) Ethyl iso-allocholate	2	Try62 → (OH)	H-bonding	1.78	− 3.0760	1.4081
		Arg128 → (OH)	H-bonding	1.86		
(20) 1,2,3-benzenetriol	1	Gln74 → (OH)	H-bonding	2.20	− 3.9130	1.1660

## Discussion

Plant extracts usually combines of various safe, and biodegradable essential oils (EOs) active ingredients may use as pests and disease control that killing harmful pests effectively. Merely 5% of pesticides worldwide are biopesticides, despite their advantageous effects as insecticides^57^. However, due to their special qualities that promote their application, such as their non-toxicity to the environment, biopesticides are growing quickly and are predicted to surpass chemical pesticides in the near future at an average annual growth rate of 9–20%^58–60^.

All the *Acacia* plant extracts that were evaluated in this study demonstrated a high level of insecticidal efficacy against mosquito larvae. Acacia methanol extracts killed mosquito larvae at 100%, 24 and 48 h post-treatment, and they were more effective than Aqueous extract. Our findings corroborated those of Baeshen and Baz^35^ research on the effects of extracts from *Salix safsafs, Eucalyptus camaldulensis,* and* A. nilotica* on *Cx. pipiens* larvae. They discovered that the extract from *A. nilotica* reduced the number of female eggs and killed mosquito larvae (100% mortality). Similarly, *A. nilotica* acetone leaf extracts were found to be acutely toxic at 212.1 mg/l and chronically toxic at 144.2 mg/l by Zaitoun et al.^61^, also who referred to the ability of *A. nilotica* extracts on inhibit adult emergence and egg hatchability.

Many multifunctional active ingredients have apparently been discovered in the seeds and leaves of *A. nilotica*^62,63^. Consequently, extracts from *A. nilotica's* fruits and leaves have demonstrated potential as fungicides, bactericides, insecticides, and molluscicides^62,64^. Vivekanandhan et al.^65^ assessed the bio efficacy of *A. nilotica* seed essential oil and seed pod solvent extracts against three significant mosquito species 24 h after treatment. Strong larvicidal efficacy against *Anopheles stephensi* (LC_50_ = 5.239 mg/L), *Aedes aegypti* (LC_50_ = 3.174 mg/L), and *Cx. quinquefasciatus* (LC_50_ = 4.112 mg/l) was demonstrated by the hydrodistilled seed oil. In contrast, the smoke toxicities for *Cx. quinquefasciatus*, *Ae. aegypti, and An. stephensi* adults were 82%, 90%, and 80%, respectively^65^.

In addition to their effectiveness on mosquito larvae, *A. nilotica's* methanol and acetone bark extracts were examined using an artificial diet bioassay for their potential to inhibit the growth of melon fly (*Bactrocera cucurbitae*) larvae in their first, second, and third instars. Also, pupation and emergence percentages were reported to be markedly decreased^66^. Studies have shown that extracts from *A. nilotica* can be effective in killing mosquito larvae. These extracts can disrupt the larvae's life cycle and prevent them from developing into adult mosquitoes^14,67,68^. In addition to being highly effective against other insects^65,69^ and microbes^63,70^.

*A. nilotica* extracts have been shown to be safe in research studies, meaning they can be used in aquatic environments where mosquitoes live. To ensure they are safe for existing organisms and to see what would happen if these extracts were used extensively, we evaluated these extracts on some non-target aquatic organisms common in mosquito larval habitats. It was found that there were no significant differences (*P* > 0.05) when applying aqueous extracts to predators, whether with aqueous or ethanol extracts.

Three of the non-target aquatic biocontrol agents were less hurt after being treated with *A. caesia* extracts and Ag NPs than mosquito larvae. The *A. caesia* LC_50_ ranged from 684 to 2245 μg/ml. Overall, our study shows that *A. caesia* has only a moderate effect on non-target aquatic biocontrol agents like *A. bouvieri, D. indicus*, and *G. affinis* fishes^71^. Specifically, the literature articles discussing the effects of the extract from the *Acacia* plant on fish or non-target organisms are rarely. However, generally speaking, a large number of literary articles support the idea that medicinal plants are not toxic to fish or non-target organisms. Promsiri et al.^72^ found that the guppy fish (*Poecilia reticulata*) was a common non-target organism found in *Ae. aegypti* habitats at levels that were effective at killing mosquito larvae. This is an example of this. They discovered that extracts from 112 different medicinal plant species showed no or very low toxicity to guppy fish. In another study that conducted the toxicological effect of *Plumbago zeylanica* and *Cestrum nocturnum* plant extracts against the fish species *P. reticulata*, which are the most widespread non-target organisms in *A. aegypti* environments, almost no significant harm was reported at doses of LC_50_ and LC_90_^73^.

As an attractive point, the descending concentration gradient leads to a descending rate of oviposition and hatching rates. At high concentrations of *A. nilotica* extract, the oviposition rate recorded its maximum value, and more than 50% of the total eggs were laid. Similarly, oviposition attitude was observed at moderate concentration, and less than 31% of the total eggs were counted. In agreement, the use of a very low concentration of *A. nilotica* showed a noticeable downfall of the number of eggs laid to less than 13% and 5% of the total eggs laid for the concentrations 250 ppm and zero (control without extract), respectively, confirming the direct relationship and oviposition rate. Moreover, the hatching rate is inconsistent with the oviposition rate. The hatchability rates were affected by the concentration gradient, and at zero concentration, the hatching ability was raised to 95% of the eggs laid at this concentration, whereas the higher concentration revealed a lower hatchability rate, confirming the inverse relationship between the concentration and the hatching rate. Based on the experimental data, the concentration of *A. nilotica* extract is directly proportional to oviposition rate and inversely proportional to hatching ability, which may introduce a reasonable interpretation for the dual benefit of acacia. Through what some active ingredients do to attract insects^74,75^, perhaps through extracting odorant chemical code, at the same time, the rest of the active ingredients help kill the larvae and reduce the chances of eggs hatching^76^.

Bioactive plant chemicals, bacteria, fungus, viruses, and protozoa are natural sources of bioinsecticides, as are pheromones and microorganisms. According to their source, the four main classes of bioinsecticides are phytochemicals, plant-incorporated protectants, microbiological pesticides, and pheromones^77^. They have been successfully used to manage and control pests^78^. Because they are more potent at very low doses, being biodegradable, less toxic, and target-specific than synthetic compounds, they are superior to them.

Plants are biological factories that generate a wide range of compounds referred as secondary metabolites. The main pharmacological effects of all medicinal plants are attributable to those secondary metabolites, which include alkaloids, carbohydrates, flavonoids, saponins, tannins, and terpenoids. Several different types of bioactive compounds that are isolated from plants or added to botanical pesticides may have negative effects on pests or other species that eat them or come into contact with them^79^.

The study examined the organic compounds present in the methanol and aqueous extracts of *A. nilotica*. The results indicated that the methanol extract had a greater variety of organic compounds, including terpenes, steroids, polyethylene glycol, and fatty acid compounds, whereas the aqueous extract had a higher concentration of fatty acid contaminants. The extracting solvents’ polarity had an impact on the antioxidant qualities, phytochemical content, and extraction yield^80^. Different extraction yields were obtained with different solvents. This is because the plant materials include high quantities of polar chemicals that are soluble in high polarity solvents, which might lead to a considerable fluctuation in the quantity of bioactive compounds in the extract due to changes in the polarity of the solvents. The findings showed that acetone contained more phytochemicals than aqueous extract, including phenols, terpenes, flavonoids, alkanes, and ketone^81^.

We find that depending on the type of solvent used in extraction and the presence of the *Acacia* plant in a specific area with specific climate, a clear variation appears in the secondary compounds. According to Edriss et al.^68^, the *A. nilotica* water and ethanol extracts revealed a broad spectrum of secondary metabolites, primarily alkaloids, saponins, flavones, and tannins, but the petroleum ether extracts were primarily composed of triterpenes and sterols. Aqueous and alcohol extracts of the plant A. nilotica were subjected to phytochemical screening. *Acacia nilotica* fruit showed the existence of phenol, terpenoid, flavonoid, reducing sugar, steroid, and flavonoids^82^.

*A. nilotica* contains various bioactive compounds, including tannins, alkaloids, and flavonoids. These compounds are believed to be responsible for its larvicidal activity, where in the Table 4 there are some of the active ingredients that are used in medical treatments, such as Methoxyphenyl piperazine (Piperazine).

Piperazine and its derivatives have attained a unique position as imperative pharmacophores in several therapeutic areas. Researchers have always been interested in using piperazine molecules as a building block to make new drug molecules that have a lot of different biological activities^83^ because of their flexible structure. Neophytadiene is a good analgesic, antipyretic, anti-inflammatory, antimicrobial, and antioxidant compound^84^.

The mission of carrying lipids and poorly soluble molecules such as retinoids, fatty acids and bile acids in addition to all hydrophobic constituents transported by the cytosol for variant purposes of storage or even utilization, is one of the intracellular binding proteins’s roles in the living biology. Such proteins are a family of low-molecular weight and single chain polypeptides binds to the hydrophobic components, from different origins, forming a matrix of complex capable of carrying poorly soluble compartments into cytosol or any organelles in the cell, this complex keep the sensitive poorly soluble components shielded from decomposition. Fatty acid binding proteins, FABPs, aids to synthesize phospholipids, lipid metabolism and mitochondrial beta oxidation furthermore a large number of functional roles of FABPs have been proposed based on physiological disorder in both animal and insect kingdoms^85^. FABPs usually expressed largely in both vertebrates and invertebrate^86^. FABPs classified into main categories in mammels constrained epidermal (E-FABP), intestinal (I-FABP), brain (B-FABP), haert (H-FABP), myelin (m-FABP), testis (T-FABP), retina (FABP12) and liver FABPs^87^. Majority of FABP proteins share high amino acid sequence similarities more than 70% however, their three-dimensional structure was highly constrained β-barrel structure with ligand binding cavity^88–90^.

The first FABP discovered was desert flight muscle locust of *Schistocerca gregaria* and Haunerland et al.^91^ followed by two abundant MFB1 and MFB2 were isolated from the midgut of Manduca sext larvae^92^. FABPs from different and variant insects heavily affects insect physiological metabolism by regulating intracellular fatty acids, it regulates sleep and long term memory consolidation, affects lipid accumulation in addition to their role in nutritional exploitation and caste division of many insects^93^. The change in FABPs chemistry will promote extensive physiological and behavioral attitude of insects. Hereon we tried to introduce a rationalized mode of action describing the activity of acacia extract, and how it affects *Cx. Pipiens*, taking the advantages of the great structure similarity of their fatty acid binding proteins (FABPs) for most insects. We used *lm-FABP* (PDB code:2FLJ) as model to evaluate each single component of the activated ingredients in acacia extract and how it binds to the target FABP.

Qualitatively, the docking results of most of the activate ingredients detected by the GC/MS were found to be close to the positive control, OLA, Due to the structure similarity between most of detected compounds and OLA. The results divided into four main categories the first one is some of tested compound revealed the same number of bindings with great similarity of amino acids residues like compound 1, methoxyphenyl piperazine, and compound 4, hexadecenoic acid, each one on them revealed three dipole–dipole interactions. The second category was recognized with candidates bonded with two hydrogen bonds like compounds (z,z,z)-9,12,15-octadecatrienoic acid [6]; 7,10,13-eicosatrienoic acid, methyl ester [8]; 9,12-Octadecadienoic acid (z,z)-, methyl ester [16]; Stearic acid methyl ester [17]; Cis-11-eicosenoic acid [18] and Ethyl iso-allocholate [19]. The nature of hydrogen bond shares the same properties of length, they have comparable length, and with the same amino acids’ residues e.g., the ester group in compound 8 interacted with Try130 and Arg128 amino acids, this is quite similar to the reference drug OLA.

**Figure 8:**
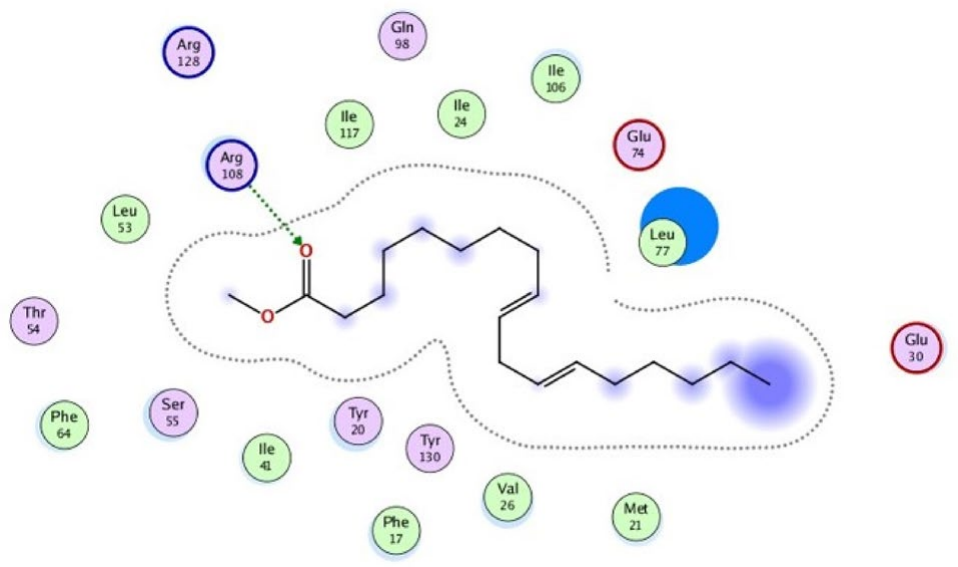
Ligand-Docking and two-dimensional receptor interactions of compound **15** (9,12-Octadecadienoic acid (z,z)-, methyl ester) interior **2FLJ** pocket.

Similarly, compound 18 and 19 revealed the same number of interactions. As shown in Fig. 8 (supplementary file) compound 18 has relatively high and not preferable RMSD. Such value determines whatever the tested ligand could simulates the co-crystallized ligand or not. Energetically the accepted value of RMSD should not exceed 1.7 and higher values are energetically not favored^41,42^. On the other side, a group of six candidates of 10, 11, 12, 13, 15 and 20 showed a smaller number of bonds however some of these compounds interacted with the same residual amino acid as the reference drug e.g., compound 10. In addition to RMSD values, the scoring energy is a very beneficial physical property and all the selected poses revealed negatively charged scoring energy. The larger the negative value of the scoring energy becomes, the more stability it indicates, and for this compounds 8 and 17 have the lowest scoring energy of -7.4504 and -7.3073 kcal/mol, respectively.

**Figure 9:**
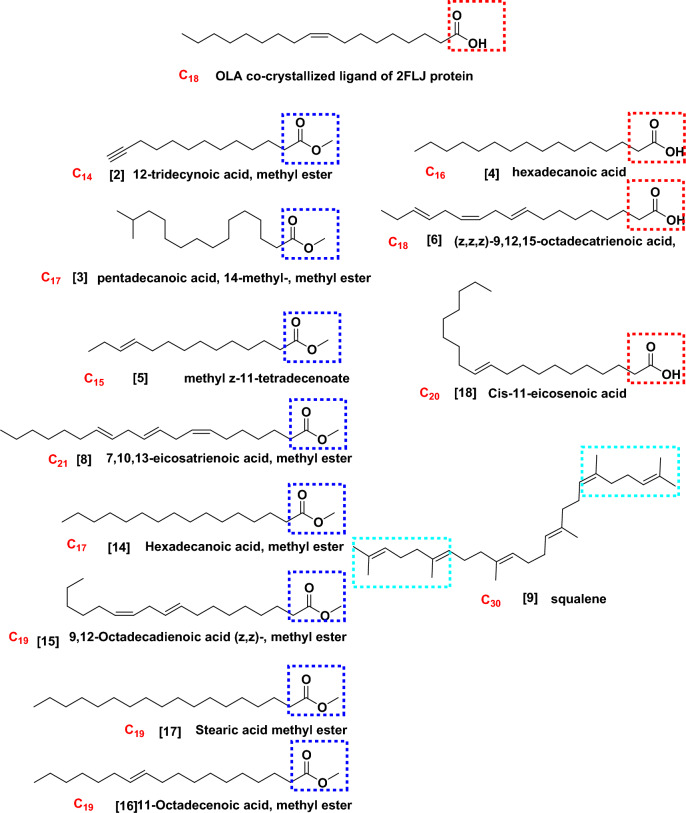
**C**hemical structure of the most active and abundant ingredients of open chain acid and esters of acacia extract compartments and the structure similarity relationship compared to the co-crystallized ligand, OLA.

From the docking results, most of the tested structures such as 2, 4, 5, 6, 8, 15, 16 17 and 18, showed at least one interaction resemble to the OLA. The most effective factor is the structure of that compounds and their structure-resemblance with oleate (OLA) co-crystallized ligand. Such compounds have long hydrocarbon chain prolonged from 14 to 20 carbon atoms (Fig. 9) and the reference drug consist of exactly 14 carbon backbone. Such resemblance makes the size of tested ligand fitting to the protein receptor pocket and that interprets the low RMSDs which strengthen the idea of compatibility. Additionally, the carboxylic (COOH) group and /or ester group (COOMe) presented in the tested compounds structurally agreed with the carboxylate (COO^–^) terminated the OLA.

**Figure 10:**
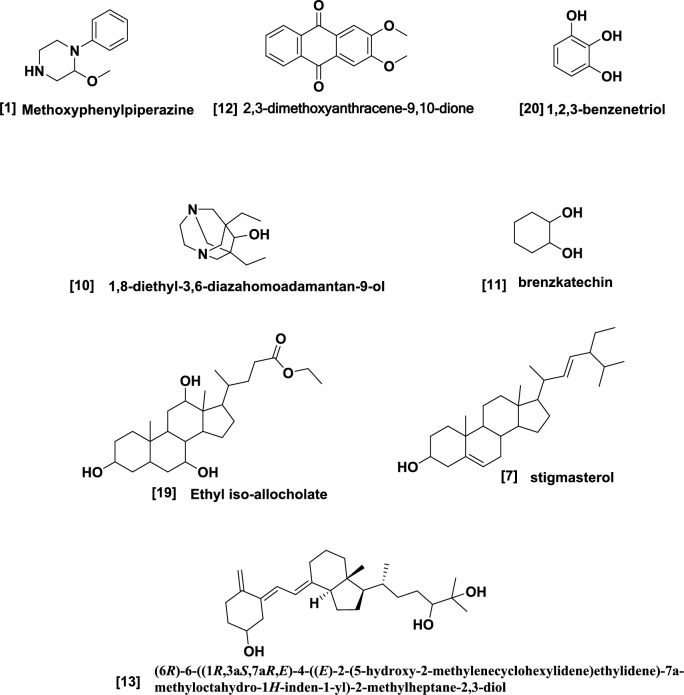
chemical structure of the most active and abundant activate ingredients of polycyclic components of acacia extract.

Extending carbon backbone more over e.g., squalene, even if it contains active part like multiple double bonds, but the size of the structure becomes non-compatible to the receptor pocket. Replacing the long chain hydrocarbon with polycyclic moieties as showed in Fig. 10 such as compounds 7 and 13 did not achieve the same purpose of binding ability.

## Conclusion

Molecular hybridization and docking simulation were used to introduce a convenient mode of action and to suggest how is acacia extracts affects *Cx. pipiens* via studying the binding compatibility of each single component detected by the GC/MS to the FABP, as one the effective proteins the invasively affect not only *Culex* but all vertebrate and invertebrates. Three components manifested unique binding ability like OLA co-crystallized ligand. Some other candidates presented exceptionally good binding abilities with higher chances of replacing OLA. The activity prediction by simulation keeps the ways smoothed for the researchers to continue exploring the potential of *A. nilotica* and its extracts as a natural and sustainable means of controlling mosquito populations and reducing the spread of mosquito-borne diseases. However, the effectiveness of these extracts may vary depending on factors such as concentration, application method, and local environmental conditions, so further research and testing are necessary to refine and optimize their use.

### Supplementary Information

Supplementary Figures.

## Data Availability

This article includes all data produced or analysed during this investigation.
