# Supplementary material for: Larvicidal activity of Acacia nilotica extracts against Culex pipiens and their suggested mode of action by molecular simulation docking
Source: Sci Rep. 2024 Mar 15;14:6248. doi: 10.1038/s41598-024-56690-2 (PMC10940718; doi:10.1038/s41598-024-56690-2)
Supplement: Supplementary file 1 — Supplementary Figures. [file 41598_2024_56690_MOESM1_ESM.doc]

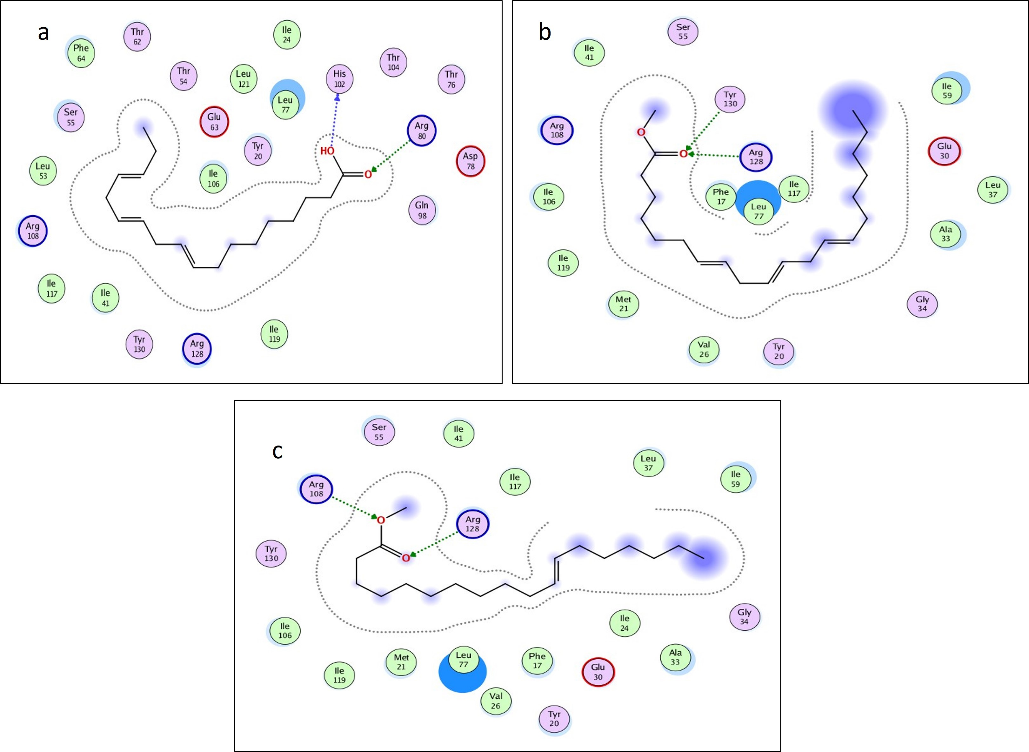


**Figure S1:** Ligand-Docking of compounds **6**, **8** and **16** interior **2FLJ** pocket: a) two-dimensional receptor interactions of compound **6** ((z,z,z)-9,12,15-octadecatrienoic acid); b) three-dimensional receptor interactions of compound **8** (7,10,13-eicosatrienoic acid, methyl ester); c) three-dimensional positioning in the receptor pocket of compound **16** (11-Octadecenoic acid, methyl ester).


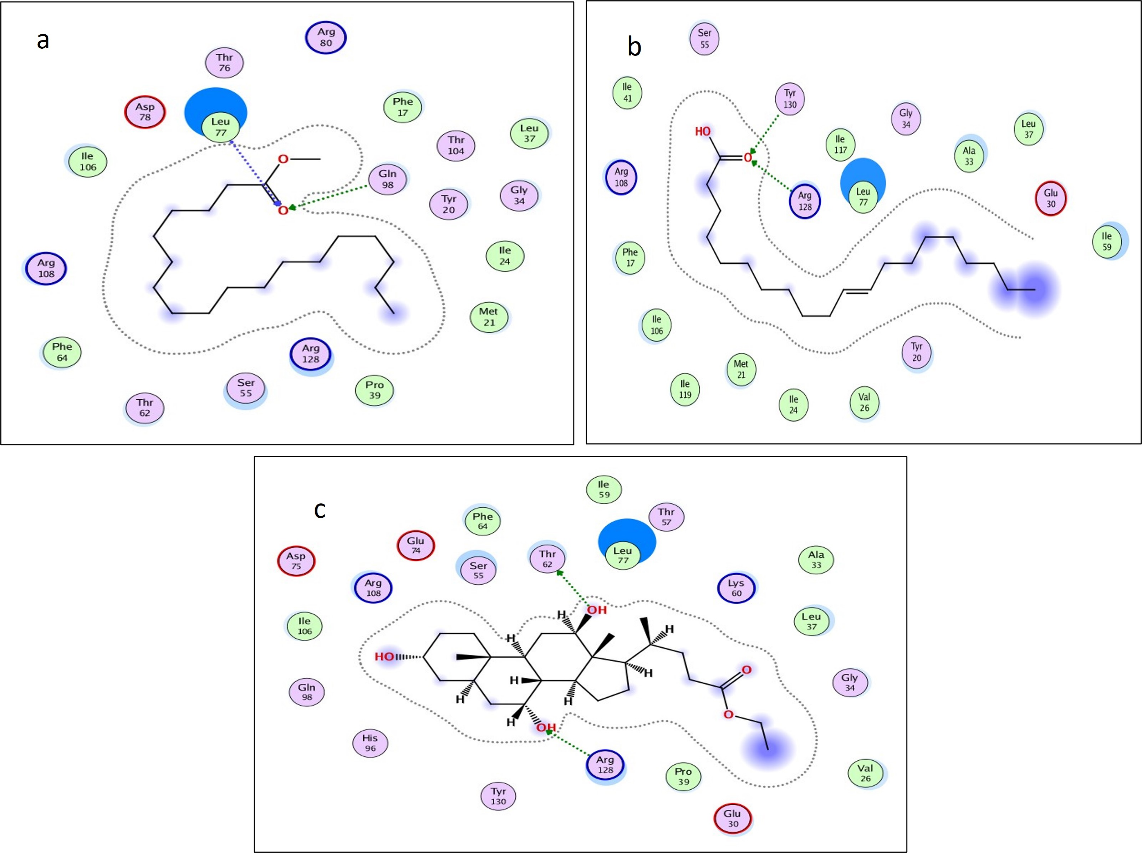


**Figure S2:** Ligand-Docking of compounds **17**, **18** and **19** interior **2FLJ** pocket: a) two-dimensional receptor interactions of compound **17** (Stearic acid methyl ester); b) three-dimensional receptor interactions of compound **18** (Cis-11-eicosenoic acid); c) three-dimensional positioning in the receptor pocket of compound **16** (11-Octadecenoic acid, methyl ester).


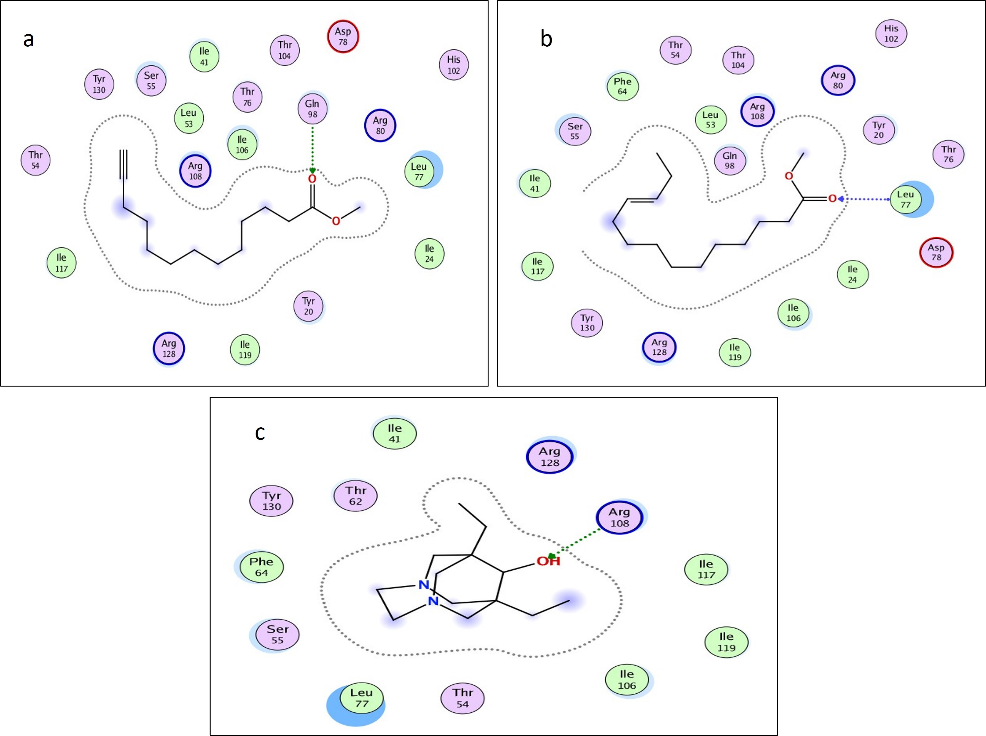


**Figure S3:** Ligand-Docking of compounds **2**, **5** and **10** interior **2FLJ** pocket: a) two-dimensional receptor interactions of compound **2** (12-tridecynoic acid, methyl ester); b) three-dimensional receptor interactions of compound **5** (methyl z-11-tetradecenoate); c) three-dimensional positioning in the receptor pocket of compound **10** (1,8-diethyl-3,6-diazahomoadamantan-9-ol).


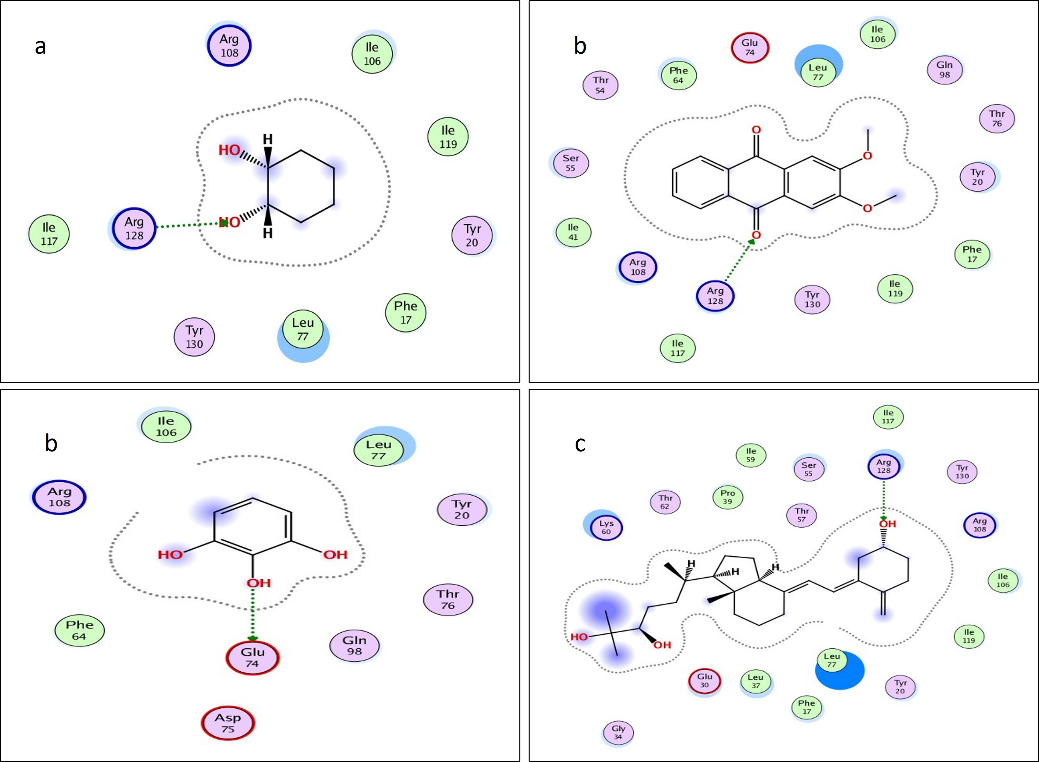


**Figure S4:** Ligand-Docking of compounds **11**, **12**, **13** and **20** interior **2FLJ** pocket: a) two-dimensional receptor interactions of compound **11** (Brenz catechin); b) three-dimensional receptor interactions of compound **12** (2,3-dimethoxyanthracene-9,10-dione); c) three-dimensional positioning in the receptor pocket of compound **13** (9,10-secocholesta-5,7,10(19)-triene-3,24,25-triol, (3á,5z,7e)); d) three-dimensional positioning in the receptor pocket of compound **20** (1,2,3-benzenetriol).


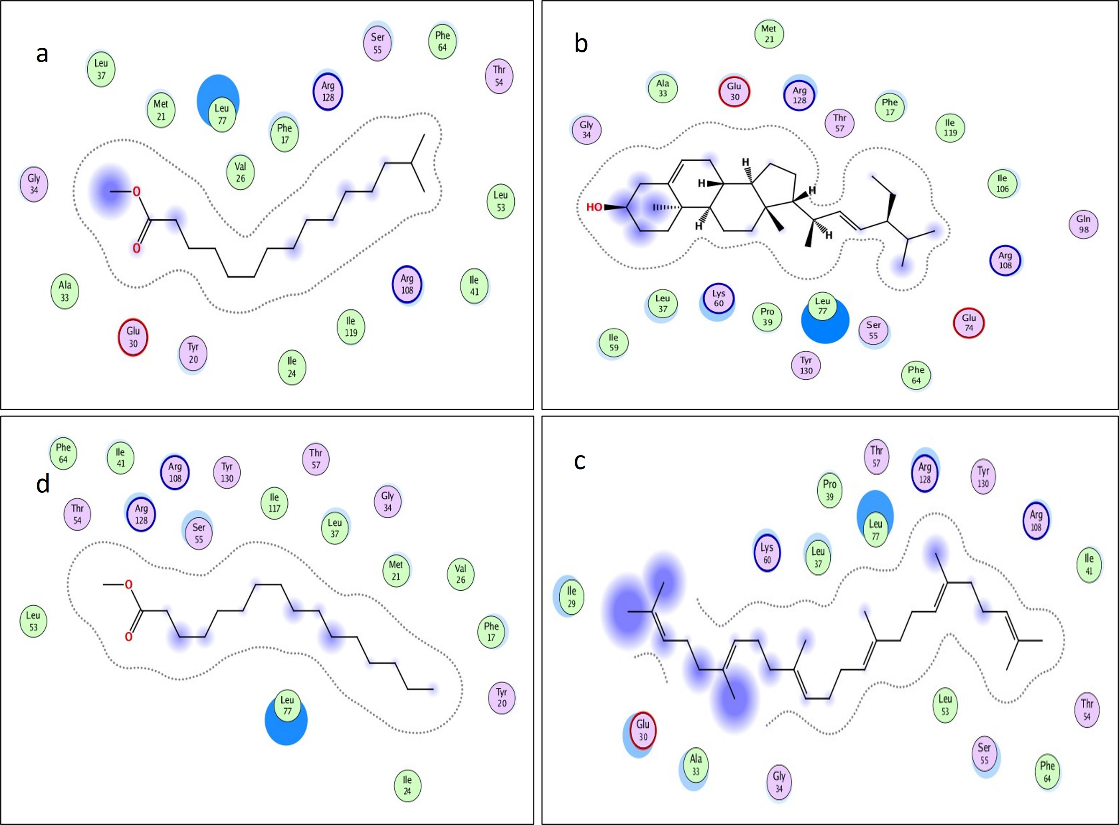


**Figure S5:** Ligand-Docking of compounds **3**, **7**, **9** and **14** interior **2FLJ** pocket: a) two-dimensional receptor interactions of compound **3** (pentadecanoic acid, 14-methyl-, methyl ester); b) three-dimensional receptor interactions of compound **7** (stigmasterol); c) three-dimensional positioning in the receptor pocket of compound **9** (squalene); d) three-dimensional positioning in the receptor pocket of compound **14** (Hexadecanoic acid, methyl ester).
